# Effect of furrow irrigation systems and irrigation levels on maize agronomy and water use efficiency in Arba Minch, Southern, Ethiopia

**DOI:** 10.1016/j.heliyon.2023.e17833

**Published:** 2023-07-04

**Authors:** Tigabie Setu, Terhas Legese, Geteneh Teklie, Birara Gebeyhu

**Affiliations:** a Water Resources and Irrigation Engineering Department, KIoT, Wollo University, Ethiopia; b Water Resources and Irrigation Engineering Faculty, AWTI, Arba Minch University, Ethiopia

**Keywords:** Deficit irrigation, Furrow irrigation, Water use efficiency

## Abstract

Improved irrigation water management is the main strategy to improve water use efficiency in areas with limited water resources. Optimizing scarce water by selecting suitable furrow irrigation systems in arid and semi-arid parts of Ethiopia is rarely practiced. A field experiment was carried out to evaluate the effect of conventional, alternate, and fixed furrow irrigation systems with three levels of irrigation (100%ETc, 75%ETc, and 50%ETc) on maize agronomy and water use efficiency in Arba Minch, Southern, Ethiopia. The experimental study had nine treatments under a randomized complete block design with three replications. Furrow irrigation system and deficit irrigation levels had a significant impact (P < 0.01) on maize agronomy, grain yield, and water use efficiency. The maximum grain yield of 7.99 ton ha^−1^ obtained under a conventional furrow irrigation system at 100%ETc was significantly higher than the other treatments and the minimum grain yield of 4.24 ton ha^−1^ obtained under a fixed furrow irrigation system at 50%ETc was significantly inferior as compared to other treatments. The maximum values of crop, field, and expense water use efficiencies were 2.49, 2.80, and 1.72 kg m^−3^ under conventional furrow irrigation at 50%ETc, respectively. The minimum values of crop, field, and expense water use efficiencies were 1.47, 1.28, and 1.05 kg m^−3^ under a fixed furrow irrigation system at 100%ETc, respectively. A conventional furrow irrigation system at 50%ETc can increase 0.64 ha net additional irrigable land pre each hectare compared to the conventional furrow irrigation system at 100%ETc. Water saving up to 50%ETc in conventional furrow irrigation can solve the water shortage problem by improving water use efficiency with insignificant yield reduction.

## 1 Introduction

Irrigated agriculture plays a significant role in growing crops, maintaining the landscape, and revegetating disturbed soils in dry areas during periods of less rainfall. It provides the livelihood of an enormous part of the world's population and supplies a large portion of the world’s food. Agriculture, which uses approximately 70% of the world’s freshwater withdrawals for irrigation, is the largest consumer of water resources globally [1]. This production sector is made by surface methods of irrigation, especially by furrow irrigation systems, which account for 97.8% of farmer’s fields and the majority of commercial farms [2]. Furrow irrigation is the most widely used surface irrigation method for water application to cropped fields. Such type of irrigation is commonly used in arid and semi-arid areas to supply water for crops and required a smaller initial investment, no more effort is required to keep improving its management and efficiency [3].

Deficit irrigation practices are an optimization strategy that intentionally allows crops to sustain some degree of water stress with less tradeoff yield and a significant reduction of irrigation water application in arid and semi-arid regions. This strategy involves manipulating the soil water to induce the crop's inherent response to drought conditions, usually to improve water use efficiency. Water use efficiency is gaining importance, particularly in arid and semi-arid regions to improve water management practices. In these regions, irrigation is required for almost all crop cultivation, and furrow irrigation is the principal means of applying irrigation water for crop production [4].

Maize (*Zea mays* L.) is an important staple grain crop for smallholder farmers in Ethiopia, and indifferent sub-Saharan countries that are cultivated under a furrow irrigation system [5]. Maize is cultivated mostly at lower altitudes along the western, southwestern, and eastern peripheries of Ethiopia. Nowadays, maize is competing for land and water with wheat and teff in the high and mid-altitudes. It is a very important crop for the preparation of local foods like Fossae, Kita, and Injera consumed as homemade drinks such as Bordie and Tella in addition used as fodder for livestock. In many parts of Ethiopia, maize is grown predominantly as a single crop and sometimes intercropped with beans using the main rainy season. The production of maize in the dry season using irrigation is also familiar in the rift valley of Ethiopia [6]. However, the production and productivity of maize crops are strongly challenged by the rapidly dwindling water resources and the growing increase in the competition for water. There is a gap in water use efficiency improvement and the critical moisture deficit level for optimal production which can help to face the challenge that occurs due to water shortage. In order to allocate scarce water resources among competing users, identifying furrow irrigation methods and irrigation levels which maximize crop water efficiency using available water was obligatory work. However, in the study area, there was no conducted research to improve the water use efficiency of maize under different furrow irrigation systems. This experiment is, therefore proposed and executed to investigate the effect of furrow irrigation systems and irrigation levels on water use efficiency of irrigated maize in Arba Minch, Southern, Ethiopia agro-climatic zone.

## 2 Materials and methods

### 2.1 Experimental site and climate

**Fig. 1:**
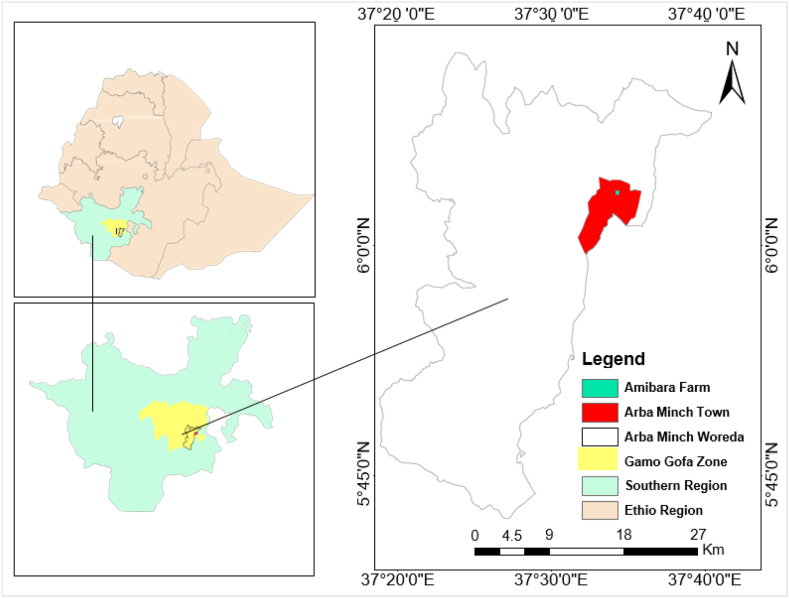
Location map of the study area.

The field experiment was conducted in Southern Nations Nationalities and Peoples Regional State, Gamo Zone, Arba Minch Zuria Woreda, in Arba Minch at Amibara demonstration farm. The area was geographically located at 6°05′ N, Latitude and 37°35′ E, Longitude at an average altitude of 1212 m a.m.s.l and situated at a distance of 454 km from the south of Addis Ababa as shown from Fig. 1.

**Fig. 2:**
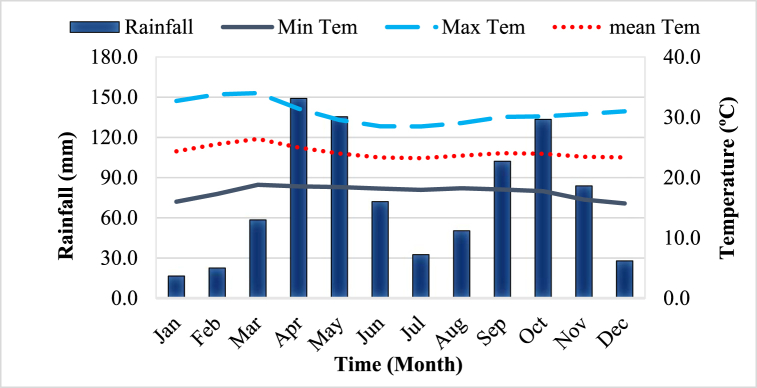
Long-term mean monthly rainfall and temperature of the study area (2005–2019).

The historical rainfall data shown in Fig. 2 is a two-season rainfall with the first and the second rainfall from April to June and September to November respectively. The average annual rainfall of the study area is about 884.5 mm. The average annual minimum and maximum temperature of the study area varies between 15.7 °C and 34.0 °C. The maize growing period is December to March, which is characterized by dry winter. Weather data for the crop growing period were obtained from the nearby weather station less than 500 m from the experimental plot.

### 2.2 Soil physiochemical properties

**Fig. 3:**
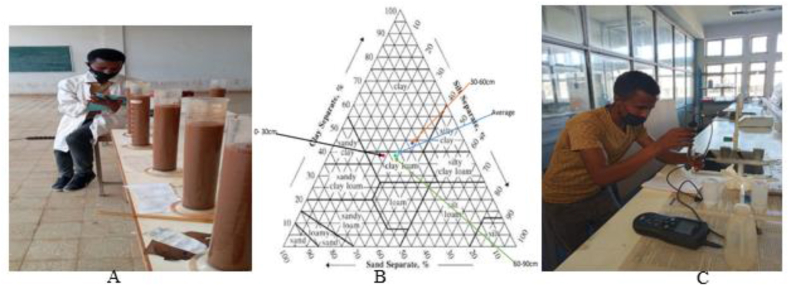
Hydrometer reading (A), soil textural triangle (B), and soil pH and (EC) reading (C).

For soil physical and chemical properties analysis, three composite representative soil samples were collected from the experimental soil up to 90 cm depths with 30 cm intervals. The 90 cm soil depth was decided based on the average root zone depth of the matured maize crop. The analyzed soil physiochemical properties were soil texture, bulk density, moisture content, infiltration rate, field capacity, permanent wilting point, the potential of hydrogen (pH), and electrical conductivity (EC). Soil texture and bulk density were evaluated by using a hydrometer test after being dispersed by sodium Meta phosphate detergent at Arba Minch University soil mechanics laboratory as shown in Fig. 3. Soil infiltration test was measured using a double-ring inflitrometer at the in-situ level and field capacity and permanent wilting point were determined in the laboratory using pressure plate apparatus. In addition to that for soil chemical properties analysis, pH and electrical conductivity (EC) of soil were determined by preparing a mixture of 1:2.5 (100 g of soil: 250 ml of distilled water) with well stirring for 30 min at room temperature [7].

Irrigation water quality was checked before being supplied to irrigated fields since irrigation water is the main source of soil salinity in addition to the upward movement of groundwater [8]. For the irrigation water quality test, water pH and salinity in terms of electrical conductivity were measured using pH meter as shown in Fig. 5 and the water sample was taken from the source canal using clean bottles.

### 2.3 Experimental design, unit plot, and culture

Table 1 shows the experiment procedures conducted for conventional (CFI), alternate (AFI), and fixed (FFI) systems combined with three levels of irrigation (100%ETc, 75%ETc, and 50%ETc).

**Table 1:** Treatment coding, furrow irrigation systems, and levels of irrigation.

Treatments	Furrow systems	Level of irrigation as % ETc
**T1**	Conventional	100
**T2**	Conventional	75
**T3**	Conventional	50
**T4**	Alternate	100
**T5**	Alternate	75
**T6**	Alternate	50
**T7**	Fixed	100
**T8**	Fixed	75
**T9**	Fixed	50

**Fig. 4:**
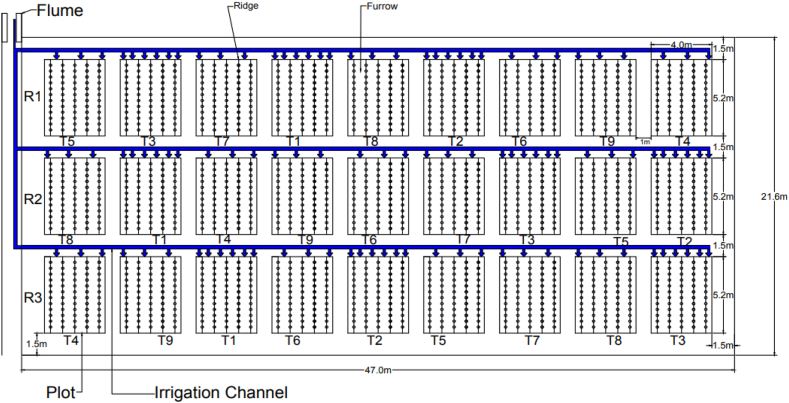
Layout of experimental plots (*T = Treatment, R = Replication).

The experimental plot location was decided in a randomized complete block design (RCBD) [9]. The size of the plots was 5.2 m × 4 m with row and plant spacing of 80 cm and 40 cm. The total experimental area was 1015.2 m^2^ (47 m × 21.6 m). There was a buffer zone of 1 m between plots and 1.5 m between blocks. Each plot had six furrows and five planting ridges as shown in Fig. 4. The central row for each treatment was considered an experimental row for data collection, and the side rows were non-experimental (buffer rows) to minimize the border effects.

The maize variety of BH-140 was selected as a test crop since is commonly grown in the area. The variety is also highly productive and appropriate for deficit irrigation [10]. Sowing was done on December 01, 2021 by sowing two seeds per hole. Thinning one plant per stand was carried out after full crop establishment. After thinning, the plant population was about 31,250 plants ha^−1^. Fertilizer was applied at a rate of 100 kg ha^−1^ and 50 kg ha^−1^ DAP (Di-Ammonium phosphate) and urea respectively. DAP was applied only at the time of sowing and urea was applied twice during the vegetative and flowering stages depending on Amibara farm cultural practice. All cultural management practices were common for all experimental treatments except the variation of furrow irrigation systems and the levels of water application levels.

### 2.4 Reference evapotranspiration, crop water requirement, and irrigation scheduling

The daily reference evapotranspiration (ETO) was determined using daily meteorological data, by applying the modified FAO Penman-Monteith equation [11] with the help of CROPWAT software 8.0.

The daily crop water requirement was determined by multiplying the reference evapotranspiration values with maize crop coefficients (0.3, 0.5, 1.2, and 0.5) at initial, development, flowering, and maturity stages respectively [11].

The net irrigation water requirement of crops (NIR) and effective rainfall (Pe) were determined after [11] as shown in Equations (1), (2).

(1) NIR=ETc−Pe−GW

Effective rainfall between irrigation events was estimated using daily crop evapotranspiration as expressed as-

(2) Pe = rainfall if, rainfall < ∑ ETc or Pe = ∑ ETc if, rainfall > ∑ ETc

where; ∑ ETc = sum of crop evapotranspiration from previous irrigation to the time of rain (mm).

The contribution of groundwater (GW) was negligible because the level of the water table (piezometric surface) recorded in the observation well near the experimental field was never closer than 2.5 m from the soil surface. Water was carefully controlled to avoid the flow into deficit plots. Since furrows are close-ended by 30 cm height bunds all water flowing into the furrow was infiltrated over the entire length, which is there was no tail water loss. Deep percolation was assumed negligible since the water was applied only to replace soil moisture in the root zone. Application efficiency was determined before the start of the actual experiment by applying the known volume of water to three selected end-blocked furrows. The Soil sample was taken before, and after 24 h water application up to the effective root depth (0.9 m) at the head, mid, and tail furrow reaches. Finally, application efficiency was determined by dividing the volume of water stored in the crop root zone by the volume of water to be applied.

The gross depth of irrigation water was determined as the ratio of the net depth of irrigation requirement to application efficiency.

The amount of water was applied at a fixed irrigation frequency of 8 days with variable depth (fill up to field capacity) and a 3-inch standard Parshall flume was set near the upstream furrows, to monitor the rate of inflowing irrigation water.

### 2.5 Measurement of irrigation water

#### 2.5.1 Non-erosive inflow discharge

It is important to determine the maximum non-erosive inflow rate before fixing the actual inflow rate, for better water management. According to Ref. [12], the maximum non-erosive inflow rate for furrows was expressed empirically using Equation (3).

(3) $$Q_{\max}=\frac{C}{S}$$

where; Q_max_ is the maximum non-erosive inflow rate (L s^−1^), S is the slope along the furrow (%) and C is the empirical constant.

The estimated maximum non-erosive inflow rate was 3 l s^−1^ by using a 0.2% average slope of the experimental plot along the furrow for clay-to-clay loam soil (0.05–0.25) after [12].

#### 2.5.2 Water application system and applied discharge

The source of water was from the Kulfo River brought upstream of the field under gravity through the earthen canal. Water is then directed to the irrigation channel by measuring using 3-inch flume. The field irrigation channel up to the furrow inlet was covered with a plastic membrane to counterbalance different losses. The irrigation water was applied per plot at a time. The rate of flow through the flume to the irrigation field was estimated as expressed by Ref. [13] as shown in Equation (4).

(4) $$Q=KH^{n}$$

where; Q is inflow rate (m^3^ s^−1^), H is inflow depth (m), and K, n are free flow constants, which depend on the size of the flume.

For the three-inch flume and the metric unit, K and n are 0.1771 and 1.55 respectively. The selected head was 5.6 cm and hence, the resulting discharge out of the Parshall flume was 2.0 l s^−1^, which was less than the maximum non-erosive inflow rate.

#### 2.5.3 Irrigation water application time

The time taken to apply irrigation water for each experimental plot was calculated as suggested by Ref. [7] as expressed in Equation (5).

(5) $$t=GIR\times \frac{A}{Q\times 60}$$

where; t is the time of irrigation (min), GIR is the gross irrigation requirement (mm), A is the area of the plot (m^2^) and Q is the inflow rate of water (l s^−1^).

### 2.6 Crop agronomy and yield measurement

**Fig. 5:**
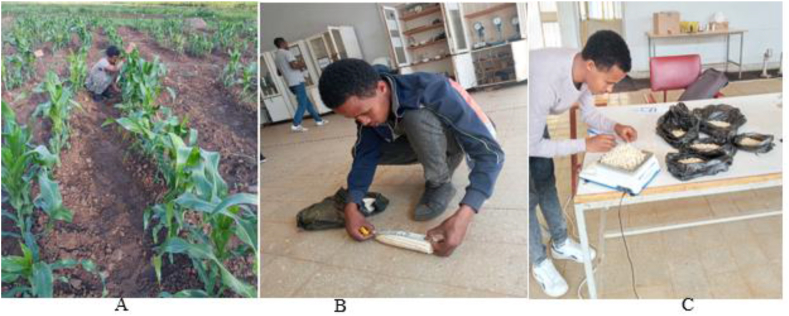
Measurement of maize (height (A), cob length and diameter (B), and grain yield (C)).

Maize agronomy and yield data collections were started three weeks after the experiment started. The data were collected from the central plant ridge at 15 days intervals throughout the cropping season in the central row of six plants in each irrigation treatment. Maize agronomy and yield recorded were plant height, days taken to tassel and silk emergence, cob length and diameter, aboveground biomass, and grain yield from each experimental treatment as shown in Fig. 5.

### 2.7 Water use efficiency (WUE) indices

The water use efficiency indices in the present study that were used to examine the performance of furrow irrigation systems and level of irrigation were crop water use efficiency (CWUE), field water use efficiency (FWUE), and field water expense efficiency (FWEE), which are expressed as suggested by Ref. [14] as shown in Equations (6), (7), (8) below respectively.

(6) $$CWUE=\frac{Y}{ETc}$$

(7) $$FWUE=\frac{Y}{GIR}$$

(8) $$FWEE=\frac{Y}{XP}$$

where; Y is crop yield (kg ha^−1^), GIR is gross irrigation requirement (mm), and Xp and water expense (mm) are expressed after [14] by using Equation (9) below.

(9) Xp=GIR+(SM1i−SM2i)×Asi×di

where; SM1i is the gravimetric soil moisture content in ith layer at the time of sowing (fraction), SM2i is the gravimetric soil moisture content in ith layer at the time of harvesting (fraction), Asi is the apparent specific gravity of the ith layer of the soil and di is a depth of soil in ith layer (mm).

### 2.8 Crop yield response factor (Ky)

The crop yield response factor (Ky) was used to quantify the relationship between crop yield and water deficit. The mathematical relationships between relative crop yield decrease to relative water deficit were used to estimate the yield response factor as proposed by Ref. [8] using Equation (10) as expressed below.

(10) $$\left(1-\frac{Ya}{Ym}\right)=Ky\left(1-\frac{ETa}{ETm}\right)$$

where; Ya is the actual crop yield (kg ha^−1^), Ym is the maximum crop yield (kg ha^−1^), and Ky is the seasonal crop yield response factor. The actual crop evapotranspiration during the experimental season was estimated from the measurement of soil moisture content. The soil moisture content was measured before and after 24 h of each irrigation event at soil depths 0–30 cm, 30–60, and 60–90 cm at each growth stage using the gravimetric method. The soil moisture measurements were made at the center of the centered furrow of each respective treatment within the plant line. ETa was estimated as expressed by Ref. [11] by using Equation (11) below.

(11) ETa=(W1−W2)×Asi×di+pe+ETca

where; ETa is actual crop evapotranspiration between irrigations (mm), W1is gravimetric soil moisture content after irrigation for ith soil layer (fraction), W2 is gravimetric soil moisture content before the next irrigation for ith soil layer (fraction), Asi is the apparent specific gravity of the ith layer of the soil, di is a depth of soil in ith layer (mm), Pe is effective rainfall between soil moisture measurements (mm) and ETca is crop evapotranspiration for one day after irrigation (mm).

### 2.9 Economic analysis

The economic analysis was done for the comparison of different treatments using existing market prices during experimentation and at the time of crop harvest. The total cost included the cost of expense for farm equipment, irrigation water, labor, fertilizer, insecticides, and maize seed. The net return was maize grain yield and saved water. The net return (NR) and benefit-cost ratio (BCR) can be calculated by using Equations (12), (13) as follows after [15] as shown in Equations (12), (13)) below.

(12) NR=GR−TC

(13) $$BCR=\frac{GR}{TC}$$

where; NR is net return, GR is gross return, TC is total cost and BCR is benefit-cost ratio.

### 2.10 Statistical analysis

The result of different maize agronomy parameters, grain yield, and different water use efficiency indices was subjected to analysis of variance (ANOVA) using R 3.6.3 software. Mean separation was carried out using the least significant difference (LSD) to compare the variation of means among treatments.

## 3 Results and discussions

### 3.1 Soil physical and chemical properties characterization

As shown in Table 2 the average determined value of soil bulk density of the experimental soil was 1.24 g cm^−3^. This value was found between 0.7 and 1.9 g cm^−3^ which was proposed by Ref. [16] for normal soil for good plant root development, aeration, water movement within the soil, and ease of uptake of nutrients by plants. According to the USDA soil textural triangle, the average soil textural class of the experimental area was clay loam with an average field capacity of 35.37, and permanent wilting point of 19.96% and an average total available soil water (TAW) was 15.41% by weight. These values were in agreement with what [17] proposed for clay loam soil.

**Table 2:** Physical and chemical properties of the experimental soil.

Soil depth (cm)	Ρb	Sand	Clay	Silt	Texture	FC	PWP	TAW	PH	EC
	(g cm^−3^)	(%)	(%)	(%)	(−)	(%)	(%)	(%)	(−)	(ds m^−1^)
0–30	1.21	37.33	38.00	24.67	CL	34.04	20.03	14.01	7.20	0.22
30–60	1.24	23.87	43.13	33.00	C	36.00	19.78	16.22	7.25	0.24
60–90	1.27	34.00	38.00	28.00	CL	36.07	20.07	16.00	7.33	0.26
Average	1.24	31.73	39.71	28.56	CL	35.37	19.96	15.41	7.26	0.24

Note: CL = clay loam, C = clay, FC = field capacity, PWP = permanent wilting point, TAW = total available water, pH = potential of hydrogen and EC = electrical conductivity.

The average soil pH value was 7.26, which was ideal for maize crop production. The ideal pH range for any soil for good plant growth ranges from 5.5 to 8 [18]. The average electrical conductivity (EC) of the experimental soil was 0.24 ds m^−1^, which was below the threshold value for maize yield reduction, i.e. 1.7 dS m^−1^. The decline in yield of maize crops starts at a threshold value of EC of 1.7 dS m^−1^ [19]. Since the EC value of soil is below the threshold value, which is safe for maize production.

The measured values of pH and electrical conductivity of irrigation water as tested in the laboratory were 7.26 and 0.24 ds m^−1^ respectively. Ref. [20] reported that for excellent irrigation water quality, the electrical conductivity should be between 0.0 and 0.80 ds m^−1,^ and the pH ranges from 6.5 to 8.4.

**Fig. 6:**
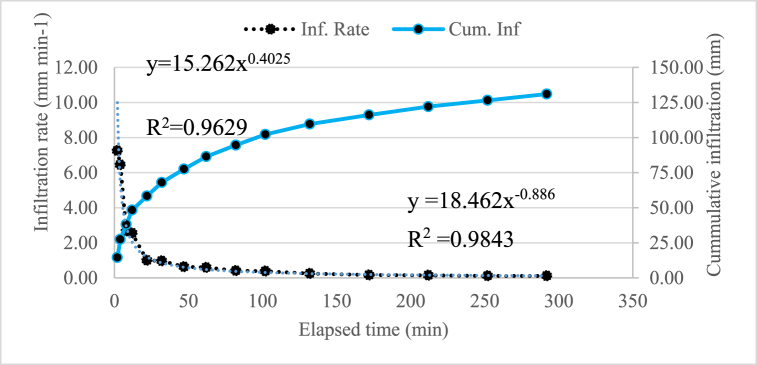
Infiltration characteristics of the experimental soil.

As shown in Fig. 6 the basic infiltration rate was 0.11 mm min^−1^. This value was found from 0.083 to 0.17 mm min^−1^ as proposed by Ref. [17] for clay loam soil.

### 3.2 Weather conditions during the crop growing period

The maize growing period, December to March, was characterized by a dry winter. The average monthly solar radiation varied from 6.78 to 9.98 h and the average monthly minimum and maximum temperatures within the crop season varied from 14.66 to 18.18 °C, and 28.12–30.96 °C, respectively. The average monthly wind speed varied from 3.93 to 47.50 km month^−1^. In addition to that, the average monthly relative humidity varied from 50.49 to 64.44% and the average monthly rainfall varied between 0.01 and 1.84 mm month^−1^ as shown in Table 3.

**Table 3:** Monthly average values of climatic data during the crop season.

	Solar radiation	Temperature (°C)		Wind speed	Relative humidity	Rainfall
Months	(MJ/m^2^/month)	Tmin	Tmax	(km month^−1^)	(%)	(mm month^−1^)
December	6.78	14.66	28.12	25.56	60.30	1.84
January	7.59	17.97	28.13	47.50	64.44	0.14
February	8.95	16.70	29.75	18.52	50.49	0.07
March	9.98	18.18	30.96	3.93	61.89	0.01

### 3.3 Crop water requirement and irrigation water application depth

**Fig. 7:**
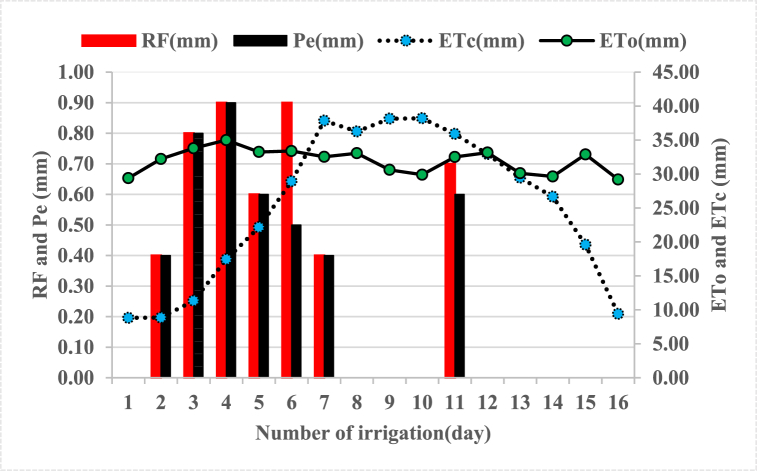
Estimated values of evapotranspiration (ETo), crop evapotranspiration (ETc), rainfall (RF), and effective rainfall (Pe) during the cropping season.

As shown in Fig. 7 crop evapotranspiration (ETc) was lower at initial and late crop growth stages and higher at development and mid-crop growing stages. The irrigation water application was important in each irrigation event since the rainfall was insufficient to cover the water needs of the crops. In cases where there is no rainfall at all during the growing season, all water has to be supplied by irrigation, in such cases; the irrigation water requirement equals the crop water requirement (ETc). Crop water requirement depends on different factors such as crop type, climate, and growth stage of the crop [11].

The estimated value of the application efficiency was found to be equal to 60.66%. The seasonal net and gross depth of irrigation were 298.75 mm and 492.52 mm, 200.76 mm, 330.87 mm, 108.51 mm, and 178.85 mm for irrigation levels of 100%ETc, 75%ETc, and 50%ETc, respectively. As indicated in Table 4 the net and gross depths of irrigation for conventional, alternate, and fixed irrigation systems at a particular level of irrigation were the same.

**Table 4:** Depth of irrigation water (mm) for each irrigation at different levels of irrigation.

	100%ETc		75%ETc		50%ETc	
Irrigation events (day)	NIR(mm)	GIR (mm)	NIR(mm)	GIR (mm)	NIR(mm)	GIR (mm)
01-Dec	8.82	14.54	6.62	10.91	4.41	7.27
09-Dec	7.81	12.88	5.60	9.23	3.39	5.58
17-Dec	8.83	14.56	6.63	10.92	4.42	7.28
25–Dec	10.14	16.71	7.61	12.53	5.07	8.36
02-Jan	10.56	17.41	5.02	8.28	5.28	8.71
10-Jan	18.08	29.81	10.85	17.88	3.62	5.96
18 –Jan	23.65	38.99	14.19	23.39	4.73	7.80
26-Jan	22.66	37.36	13.60	22.41	4.53	7.47
03-Feb	23.84	39.30	14.31	23.58	4.77	7.86
11-Feb	23.87	39.35	14.32	23.61	4.77	7.86
19-Feb	22.44	36.99	13.46	22.19	4.49	7.40
27-Feb	32.93	54.29	24.70	40.71	16.47	27.14
07-Mar	29.46	48.57	22.10	36.42	14.73	24.28
15-Mar	26.66	43.95	20.00	32.96	13.33	21.97
23-Mar	19.60	32.31	14.70	24.23	9.80	16.16
31-Mar	9.40	15.50	7.05	11.62	4.70	7.75
Total	298.75	492.52	200.76	330.87	108.51	178.85

### 3.4 Effect of furrow irrigation systems and irrigation levels on maize agronomy

#### 3.4.1 Plant height

**Fig. 8:**
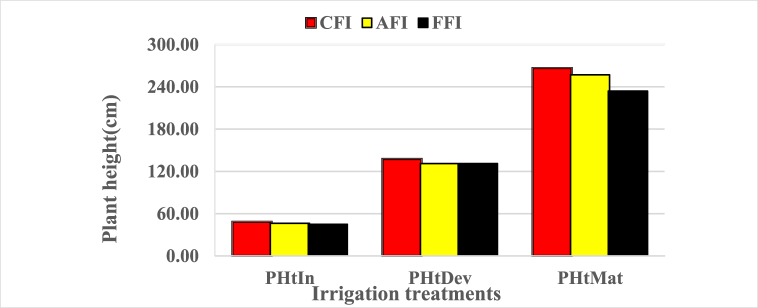
Effect of furrow irrigation systems on plant height (PHtIn = plant height at initial, PHtDev = plant height at development, and PHtMat = plant height at maturity).

The effect of the furrow irrigation system and irrigation levels on different maize agronomic attributes and grain yield for the various treatments are indicated in Table 5. As shown in Fig. 8 the plant height of 50.10, 141.23, and 280.37 cm obtained under CFI at 100%ETc at the initial, development, and maturity stages was statistically superior to all other treatments. The minimum plant height of 43.07, 124.43, and 197.37 cm observed under FFI at 50%ETc at initial, development, and maturity stages was statistically inferior to all other treatments. However, there was no significant difference in the plant height at the initial stage between CFI at 75%ETc and AFI at 100%ETc, AFI at 50%, and FFI at 75%, CFI at 50%ETc and AFI at 75%ETc. The reason to obtain maximum plant height under a conventional furrow irrigation system at 100%ETc might be full irrigation depth application and crop rows get water in each irrigation event that might lead to a higher moisture content of the soil, creating favorable growth conditions for maize. On the other hand, in the alternate and fixed furrow irrigation systems, not all crop rows get water in each irrigation event, which might lead to an effect agronomy of maize as moisture stress affect photosynthesis. Earlier workers also reported that maximum plant height was recorded in conventional furrow irrigation systems followed by alternate and fixed furrow irrigation systems [4,21,22].

**Table 5:** Effects of furrow irrigation systems and irrigation levels on maize agronomy and yield.

	Maize agronomy and grain yield									
	Plant height (cm)									
	PHtIn	PHDev	PHMat	Tassel	Silk	CL	CD	AGB	GY	LAI
Treatments	(cm)	(cm)	(cm)	(days)	(days)	(cm)	(cm)	(ton ha^−1^)	(ton ha^−1^)	(−)
CFI 100%ETc	50.10a	141.23a	280.37a	67.73a	81.04a	26.60a	20.67a	18.26a	7.99a	0.89a
CFI 75%ETc	47.32d	136.26d	260.20d	65.19c	78.72d	22.12d	17.25d	14.09c	5.50d	0.72c
CFI 50%ETc	47.10e	135.50d	259.63d	64.27d	78.34f	19.19f	15.06f	12.04d	5.01f	0.66d
AFI 100%ETc	48.23b	139.97b	271.42b	67.09 ab	80.16b	24.74b	19.65b	16.70b	7.12b	0.78b
AFI 75%ETc	47.10e	131.00e	255.00e	64.00de	78.33e	20.05e	16.05e	11.08e	5.47e	0.65d
AFI 50%ETc	45.27f	125.23g	244.93f	63.20ef	78.10g	18.78g	14.28g	8.03g	4.41h	0.61e
FFI 100%ETc	47.77c	137.70c	268.18c	66.34b	80.00c	23.90c	18.71c	15.60c	5.91c	0.73c
FFI 75%ETc	44.43g	128.13f	236.60g	62.47f	77.30h	15.21h	12.18h	8.41f	4.96g	0.59f
FFI 50%ETc	43.07h	124.43g	197.37h	60.06g	76.38i	13.29i	10.42i	7.81h	4.24i	0.51g
LSD (0.05)	1.06	1.08	1.27	0.86	0.14	0.29	0.40	0.55	0.09	0.03
CV (%)	1.31	0.47	0.29	0.77	0.1	0.81	1.44	2.55	0.94	2.53

*Means that had the same letters in the column are no significant differences at P < 0.05. PHtIn = plant height at the initial stage, PHDev = plant height at the development stage, PHMat = plant height at maturity sage, CL = cob length, CD = cob diameter, AGB = above-ground biomass, GY = grain yield, LAI = leaf area index, LSD = least significance difference and CV = coefficient of variation.

#### 3.4.2 Days to the emergence of tassel and silk

The analysis of the result reveals that the days to tassel, and silk emergence were longer as the deficit level decreases. In addition to that, the order of days to tassel, and silk emergence were fixed furrow less than alternate furrow and followed by a conventional furrow irrigation system. This might be due to that the crop rows get water in each irrigation event under the CFI system, which leads to more uniform soil moisture distribution in the root zone than the AFI system and FFI system. There was a significant difference in the date of emergence of a tassel and silk recorded in each irrigation treatment. In line with this, increasing irrigation levels leads to make favorable conditions for maize physiological and photosynthesis processes [21]. The emergence of tassel and silk are important phonological stages of maize crop development because it signs a change of growth of the crop from vegetative to generative phase, prior to yield formation. The result indicated that treatments that lacked irrigation water advanced yield maturity. This could be the fact that plants under stress tend to complete their life cycle, which enables them to escape from unfavorable conditions by ending their lifecycle a few days earlier than the normal or high soil moisture conditions, thereby ensuring the continuation of the species [23].

#### 3.4.3 Cob length (CL) and cob diameter (CD)

**Fig. 9:**
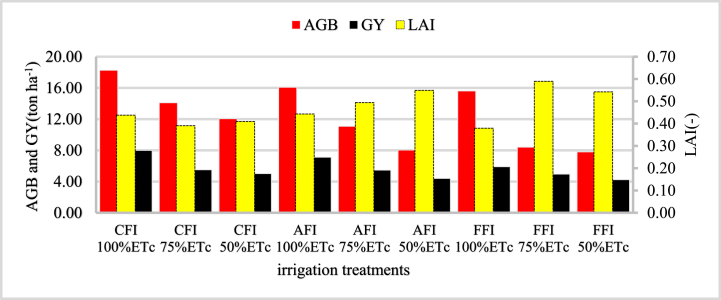
Effect of furrow system and irrigation levels on above-ground biomass (AGB), grain yield (GY), and leaf area index (LAI).

Maize cob length and cob diameter were highly significant (p < 0.01) affected by the furrow irrigation system and irrigation level. The size of the cob length and cob diameter increased as the level of irrigation increased (Fig. 9). The analysis of variance indicated that there was no statistical similarity between cob length and cob diameter under three furrow irrigation systems with different irrigation levels. Cob length and diameter recorded under conventional furrow irrigation at 100%ETc improved by 50.04% and 49.59% than cob length and diameter obtained under fixed furrow irrigation at 50%ETc respectively.

#### 3.4.4 Aboveground biomass (AGB), grain yield (GY), and leaf area index (LAI)

Conventional furrow irrigation at 100%ETc improved the aboveground biomass and grain yield of maize by 57.23% and 46.93% than obtained under fixed furrow irrigation at 50%ETc respectively. As far as water is the main component of photosynthesis for plants to produce their food, supplying adequate water might lead to an increase in above-ground biomass and grain yield. Ref. [24] reported that more than 90% of plant biomass and Ref. [23] reported approximately 70–90% of the final grain yield is derived from photosynthesis products, whose main input is water. The maximum leaf area index of 0.89 exhibited under CFI at 100%ETc was statistically superior to all other treatments, and the minimum leaf area index of 0.51 obtained under FFI at 50%ETc was statistically inferior to the rest of the treatments. The maximum leaf area index was taken at the initiation stage of flowering because the effective full cover for many crops occurs at the initiation of flowering, which was better for treatment comparison [25].

The result of the present study indicated that maize grain yield varied from 7.99 to 4.24 ton ha^−1^ which was lower and higher from investigated by different researchers. This variation in maize yield might be due to the influence of crop characteristics, irrigation methods, level of water deficit, and climatic conditions of the area [26]. The effect of the furrow irrigation system and irrigation level on maize agronomy attributes and grain yield were highly significant at (p < 0.01). Ref. [27] reported that an increase in water application depth favors the photosynthesis rate and decreases the respiration rate, which results in high dry matter production. Refs. [23,28,29] reported that maize yield and yield components were linearly related to the depth of water application.

### 3.5 Effect of furrow irrigation systems and irrigation levels on water use efficiency indices

#### 3.5.1 Crop water use efficiency (CWUE)

The effect of the furrow irrigation system and irrigation levels on different water use efficiency indices are presented in Table 6. Water use efficiency of the crop was highly significant at (p < 0.01) both by furrow irrigation systems and irrigation levels. The maximum crop water use efficiency of 2.49 kg m^−3^ obtained under CFI at 50%ETc was statistically superior for all irrigation treatments and the minimum crop water use efficiency of 1.47 kg m^−3^ recorded under fixed furrow irrigation at 100%ETc was statistically inferior for all irrigation treatments. As the level of irrigation increased, crop water efficiency decreased. However, some treatments received the full depth of irrigation but the crop water use efficiency was relatively higher such as CFI at 100%ETc. For full and three-fourth irrigation level treatments the value of CWUE was higher than FWUE and FWEE, which resulted from lower crop evapotranspiration than the gross depth of irrigation and depth of water expense, respectively.

**Table 6:** Effect of furrow irrigation systems and irrigation levels on water use efficiency.

					Water use efficiency indicators		
	ETc	Yield	Gross	Xp	CWUE	FWUE	FWEE
Treatments	(mm)	(kg ha^−1^)	irrigation (mm)	(mm)	(kg m^−3^)	(kg m^−3^)	(kg m^−3^)
CFI 100%ETc	401.91	7989.36	492.52	552.12	1.99d	1.62e	1.45b
CFI 75%ETc	301.43	5556.35	330.87	405.51	1.84e	1.68d	1.36d
CFI 50%ETc	200.96	5012.21	178.85	291.48	2.49a	2.80a	1.72a
AFI 100%ETc	401.91	7118.04	492.52	566.36	1.77f	1.45g	1.26e
AFI 75%ETc	301.43	5474.41	330.87	431.33	1.82e	1.65de	1.27e
AFI 50%ETc	200.96	4414.05	178.85	305.80	2.20b	2.47b	1.44b
FFI 100%ETc	401.91	5908.12	492.52	561.67	1.47h	1.20h	1.05g
FFI 75%ETc	301.43	4953.84	330.87	425.15	1.64g	1.50f	1.17f
FFI 50%ETc	200.96	4234.84	178.85	300.73	2.11c	2.37c	1.41c
LSD (0.05)					0.04	0.05	0.03
CV (%)					1.29	1.46	1.18

*Means which had the same letters in the column are no significant differences at P < 0.05. XP is water expense, CWUE is crop water use efficiency, FWUE is field water use efficiency, FWEE is field water expense efficiency, LSD is the least significant difference and CV is coefficient of variation.

The result also revealed that crop water use efficiency was decreased as the irrigation levels increased, and/or yield decreased, and vice versa. The reason to obtain minimum crop water use efficiency under the full depth of application might be much water lost through soil evaporation and deep percolation. On the other hand, under the conventional furrow irrigation system, maize grain yield was maximum which leads to maximized crop water use efficiency. Furthermore, crop water use efficiency and irrigation water use efficiency are influenced by crop yield, irrigation methods, and deficit level. The one that received a higher amount of water has not necessarily produced the highest crop water use efficiency; rather the one that received a lower amount of water has necessarily produced the highest crop water use efficiency. Ref. [30] reported that crop water use efficiency for maize crops ranged from 1.1 to 2.7 kg m^−3^ and crop water productivity is attributed due to climate, irrigation practice, water, and soil management.

#### 3.5.2 Field water use efficiency (FWUE)

FWUE was higher than CWUE at 50%ETc and FWEE for all irrigation levels, which resulted from lower gross irrigation depth as compared to crop evapotranspiration (ETc) and water expense. The maximum FWUE was obtained under CFI at 50%ETc due to the lesser gross irrigation depth as compared to other treatments except for AFI and FFI, at 50%ETc, the reason is that the maximum crop yield obtained under CFI at 50%ETc. The values of field water use efficiency decreased as the irrigation levels increased. The reason to obtain minimum field water use efficiency under the full depth of application might be much water was lost through soil evaporation and deep percolation. On the other hand, under the conventional furrow system, maize grain yield was maximum which weighted water deficit that leads to maximized field water use efficiency.

#### 3.5.3 Field water expense efficiency (FWEE)

**Fig. 10:**
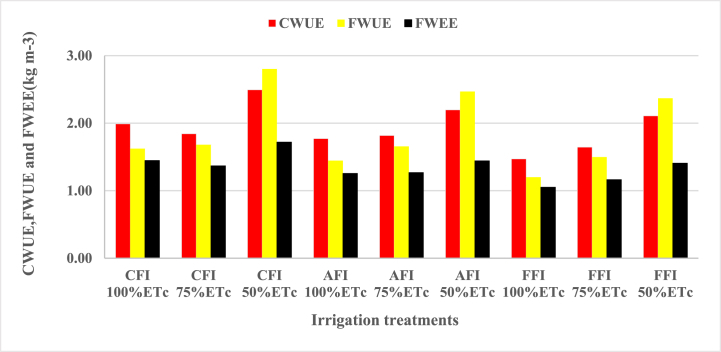
Effect of furrow irrigation systems and irrigation levels on crop water use efficiency (CWUE), field water use efficiency (FWUE) and field water expense efficiency (FWEE).

The maximum field water expense efficiency of 1.72 kg m^−3^ obtained under CFI at 50%ETc was statistically superior for all irrigation treatments and the minimum field water expense efficiency of 1.05 kg m^−3^ was recorded under fixed furrow irrigation at 100%ETc were statistically inferior for all treatments. The depth of water expense for all treatments was higher than crop evapotranspiration and gross depth of irrigation, which made FWEE lower than CWUE and FWUE for all treatments. As shown in Fig. 10 the variation of FWEE in between treatments was small. The reason might be due to the soil moisture at crop sowing being the same for all treatments but at harvesting the soil moisture for each treatment was different. The maximum soil moisture was recorded in treatments with 100%ETc with minor differences from 75%ETc and 50%ETc irrigation treatments at the time of harvesting. This maximum soil moisture for 100%ETc was deducted from the soil moisture during sowing (similar value), which results in a smaller value than 75%ETc and 50%ETc.This made the depth of water expense becomes compensate for the decreased gross depth of irrigation. Likewise, [31] reported that field water expense efficiency increased when the irrigation level decreased.

The increment of water use efficiency is directly related to either improving crop production or reducing water consumption which means at water use efficiency increased by enhancing crop yield not only depends on reducing the depth of application. The water use efficiency values obtained in this study were found between 1.05 and 2.80 kg m^−3^ slightly varied compared to the maize crop water use efficiency reported by different investigators. The variation in water use efficiency might be due to the influence of crop characteristics, irrigation method, level of irrigation, and climatic characteristics of the region [26]. In addition to that, Ref. [32] reported that not only the deficit level but also crop yield affected the water use efficiency of the maize crop thus, increased maize yield leads to increased water use efficiency besides application level reduction. Ref. [30] reviewed measured crop water use efficiency for several crops around the world, including maize, and concluded that the crop water use efficiency could be significantly increased if irrigation was reduced and crop water deficit was intentionally induced.

### 3.6 Extent of water saved and yield increment

The full irrigation water application and the maximum yield obtained under the full depth of application were used for the comparison of the corresponding treatments. Thus, the result indicated that the additional irrigable area of 0.64 ha will lead to obtaining the extra yield of 5088.16 kg ha^−1^ was obtained at 50%ETc under conventional furrow systems (Table 7). On the other hand, the minimum further irrigable area of 0.33 ha will lead to obtaining the minimum extra yield of 1939.10 kg ha^−1^ was obtained at 75%ETc under a fixed furrow irrigation system. As a result, the maximum total grain yield of 10100.37 kg ha^−1^ will be obtained under CFI at 50%ETc irrigation level and the minimum total grain yield of 5908.12 kg ha^−1^ was obtained under a fixed furrow irrigation system at 100%ETc irrigation level. Furthermore, the result indicated that treatments produced a higher amount of grain yield; there was a minimum amount of saved water, further irrigable areas, and extra yield, and vice versa. It indicates that the total yield generated was not only influenced by water applied but also by furrow irrigation systems.

**Table 7:** Extent of saved water and yield increment for different irrigation treatments.

	Applied water	Actual Yield	Water saved	Additional irrigable area	Additional yield	Total yield
Treatments	(m^3^ ha^−1^)	(kg ha^−1^)	(m^3^ ha^−1^)	(ha)	(kg ha^−1^)	(kg ha^−1^)
CFI100%ETc	4925.20	7989.36	0.00	0.00	0.00	7989.36
CFI75%ETc	3308.70	5556.35	1616.50	0.33	2622.19	8178.54
CFI50ETc	1788.50	5012.21	3136.70	0.64	5088.16	10100.37
AFI100%ETc	4925.20	7118.04	0.00	0.00	0.00	7118.04
AFI75%ETc	3308.70	5474.41	1616.50	0.33	2336.21	7810.62
AFI50%ETc	1788.50	4414.05	3136.70	0.64	4533.25	8947.30
FFI100%ETc	4925.20	5908.12	0.00	0.00	0.00	5908.12
FFI75%ETc	3308.70	4953.84	1616.50	0.33	1939.10	6892.94
FFI50%ETc	1788.50	4234.84	3136.70	0.64	3762.69	7997.53

### 3.7 Crop yield response factor (ky)

As indicated in Table 8, the highest (ky) value of 1.15 and 1.18 was attained at treatment (T8) and treatment (T9), respectively. These highest Ky values are an indication of severe water stresses which implies that the rate of relative yield decrease resulting from water stress is proportionally greater than the decrease in water deficit because of stress. The lowest (ky) values of 0.90 and 0.91 were attained at treatment (T3) and treatment (T2), respectively which indicated that the rate of relative yield decrease resulting from water stress is proportionally the same as the relative evapotranspiration. Refs. [6,33] investigated that the seasonal yield response factor (Ky) for maize crops is equal to 1.03 and 1.04 respectively. The variation in yield response factor might be due to the variation in irrigation practice, rainfall, climatic variation, and crop characteristics [28,34]. Some studies revealed that the maize crop is sensitive to water deficit. Refs. [6,33] investigated that the seasonal yield response factor (Ky) for maize crop is equal to 1.03 and 1.04 respectively and other studies found that the seasonal yield response factor for maize crop was less than unity [23,26]. The variation in yield response factor might be due to the variation in irrigation practice, rainfall, climatic variation, and crop characteristics [28,34].

**Table 8:** Seasonal water deficit and relative yield decrease for different treatments.

Treatments	ETa (mm)	Ya (kg ha^−1^)	Ym (kg ha^−1^)	ETm (mm)	1-Ya/Ym	1-ETa/ETm	Ky(−)
T1	452.15	7989.36	7989.36	452.15	0.00	0.00	0.00
T2	300.13	5556.35	7989.36	452.15	0.31	0.34	0.91
T3	266.00	5012.21	7989.36	452.15	0.37	0.41	0.90
T4	403.13	7118.04	7989.36	452.15	0.11	0.11	1.00
T5	305.15	5474.41	7989.36	452.15	0.31	0.33	0.94
T6	260.16	4414.05	7989.36	452.15	0.45	0.42	1.07
T7	349.39	5908.12	7989.36	452.15	0.26	0.23	1.13
T8	304.14	4953.84	7989.36	452.15	0.38	0.33	1.15
T9	271.37	4234.84	7989.36	452.15	0.47	0.40	1.18

Note; Ya is actual grain yield, Ym is maximum grain yield, ETa is actual evapotranspiration, ETm is maximum evapotranspiration and Ky is yield response factor.

### 3.8 Economic comparison of treatments

The estimated values of economic analysis are given in Table 9. The cost expense for all treatments was the same with only the variation of water expense. The saved water and maize grain yield were used as outputs for economic analysis. The prevailing market price of maize in the local market at the time of crop harvest was 14 Ethiopian Birr (ETB) kg^−1^ and the irrigation water price was taken as 3 ETB per 1000 m^3^ for Awash River [35].

**Table 9:** Economic analysis of maize yield production under different treatments.

Treatments	Total cost (ETB ha^−1^)	Gross return (ETB ha^−1^)	Net return (ETB ha^−1^)	Benefit-cost ratio
CFI 100%ETc	50563.84	111851.04	61287.20	2.21
CFI 75%ETc	50559.22	77793.51	27234.29	1.54
CFI 50%ETc	50554.64	70180.14	19625.50	1.39
AFI 100%ETc	50563.84	99652.56	49088.72	1.97
AFI 75%ETc	50559.22	76646.35	26087.13	1.52
AFI 50%ETc	50554.64	61805.90	11251.26	1.22
FFI 100%ETc	50563.84	82713.68	32149.84	1.64
FFI 75%ETc	50559.22	69358.37	18799.15	1.37
FFI 50%ETc	50554.64	59296.96	8742.33	1.17

The result of the economic analysis revealed that the highest net return of 61287.20 ETB ha^−1^ with the highest benefit-cost ratio of about 2.21 was obtained under the conventional furrow irrigation system at 100%ETc, which exhibited maximum grain yield production. On the other hand, the lowest net return of 8742.33 ETB ha^−1^ was obtained under the fixed furrow irrigation system at 50%ETc with a benefit-cost ratio of 1.17, which exhibited minimum grain yield production. Moreover, the conventional furrow irrigation system was more preferential in net return and benefit-cost ratio followed by alternative and fixed furrow irrigation systems.

## 4 Conclusions and recommendations

Treatments received at the full depth of irrigation under the conventional furrow irrigation system were more reactive on maize agronomy (plant height, days to tassel and silk, cob length and diameter, aboveground biomass) and grain yield, as compared to other plots*.* Plots that received half the depth of irrigation under a fixed furrow irrigation system were less responsive on maize agronomy (plant height, days to tassel and silk, cob length and diameter, aboveground biomass) and grain yield, as compared to other plots.

Adaptation and adoption of improved water management strategies in furrow irrigation systems have a pivotal role in the improvement of water use efficiency in areas where water resources are limited. The result of the study indicated that higher water use efficiency was obtained under the conventional furrow irrigation systems at different levels of irrigation water application as compared to alternate and fixed furrow irrigation systems. Among all irrigation treatments, the 50%ETc deficit level applied under the conventional furrow irrigation system was efficient in conserving significant irrigation water at the same time attaining optimum yield. It could be recommended that increased water saving and water use efficiency through irrigation at a 50%ETc deficit level under conventional furrow irrigation would ensure the scope of further irrigation development.

## Author contribution statement

Tigabie Setu Birhan; Terhas Legese Beyene; Geteneh Teklie Alemu; Birara Gebeyhu Reta: Conceived and designed the experiments; Performed the experiments; Analyzed and interpreted the data; Contributed reagents, materials, analysis tools or data; Wrote the paper.

## Data availability statement

Data will be made available on request.

## Additional information

No additional information is available for this paper.

## Declaration of competing interest

The authors declare that they have no known competing financial interests or personal relationships that could have appeared to influence the work reported in this paper.
